# Daily Cognitive Difficulties and Social Experiences Among Older Adults

**DOI:** 10.1093/geroni/igab046.2599

**Published:** 2021-12-17

**Authors:** Ruixue Zhaoyang, Jacqueline Mogle, Karra Harrington, Martin Sliwinski

**Affiliations:** 1 The Pennsylvania State University, University Park, Pennsylvania, United States; 2 Penn State University, University Park, Pennsylvania, United States

## Abstract

Self-reported cognitive difficulties are common in older adults and may be an early indicator of future cognitive decline or dementia. In past retrospective reports, cognitive difficulties have been linked with differences in social engagement or social relationships among older adults. However, little is known about how self-reported cognitive difficulties in daily life, such as memory lapses, relate to older adults’ daily social experiences. This study examined how self-reported cognitive difficulties were related to older adults’ daily social interactions and loneliness. Data were drawn from 312 community-dwelling older adults (aged 70 to 90 years) who reported their social interactions and loneliness throughout the day (five times) as well as cognitive difficulties (e.g., memory lapses, problems with attention) at the end of each day for 14 days. Multilevel models revealed that participants reported fewer memory lapses on days when they reported more frequent interactions with family members (p=.041). Higher levels of disruptions to daily activities caused by cognitive difficulties, in turn, predicted higher levels of loneliness the next day (p=.006), but not changes in social interactions the next day. At the between-person level, more memory lapses in daily life were associated with less frequent social interactions with friends, but more frequent unpleasant social interactions and higher levels of loneliness on average. These results suggest that older adults’ self-reported cognitive difficulties were dynamically associated with their social interactions and loneliness at the daily level and played an important role in older adults’ social life and well-being.

